# Synergistic effects of IL-4 and TNFα on the induction of B7-H1 in renal cell carcinoma cells inhibiting allogeneic T cell proliferation

**DOI:** 10.1186/1479-5876-12-151

**Published:** 2014-05-30

**Authors:** Dagmar Quandt, Simon Jasinski-Bergner, Ulrike Müller, Bianca Schulze, Barbara Seliger

**Affiliations:** 1 Institute of Medical Immunology, Martin Luther University Halle-Wittenberg, Magdeburger Str. 2, Halle 06112, Germany

**Keywords:** Renal cell carcinoma, Costimulation, T cells, Anti-tumor response, B7-H molecules

## Abstract

**Background:**

The importance of B7-H molecules for the T cell/tumor communication and its impact on renal cell carcinoma (RCC) progression and prognosis has been recently described. Cytokine treatment of RCC has earlier been shown to be beneficial in preclinical settings, but its clinical implementation has not proven to be as effective. This might be partially explained by the yet incomplete picture of cellular alterations in tumor cells upon cytokine treatment investigated in detail in this study.

**Methods:**

RCC tumor cell lines were treated with different cytokines alone or in combination. The constitutive and/or cytokine-induced expression of cytokine receptors signaling components and B7-H molecules in RCC cells were analysed by qPCR and flow cytometry. A mcherry reporter gene construct containing B7-H1 promoter was cloned and its activity was determined upon transfection in cytokine-stimulated cells. Cytokine pretreated tumor cells were co-cultured with allogeneic CD8^+^ T cells from healthy donors and T cell proliferation as well as cytokine secretion was determined.

**Results:**

A heterogeneous, but constitutive B7-H1,-H2,-H3 and H4 expression was found on human RCC cell lines. IL-4 and TNFα treatment led to strong synergistic induction of B7-H1 in RCC cells, whereas B7-H2 was only increased by TNFα. In contrast, B7-H3 and B7-H4 expression were not altered by these cytokines. Treatment of RCC cells with TNFα and IL-4 was accompanied by an activation of signaling molecules like NF-κB, IκB and STAT6. The cytokine-mediated up-regulation of B7-H1 was due to transcriptional control as determined by an increased B7-H1 promoter activity in the presence of IL-4 and TNFα. Despite HLA class I and LFA-1 were also increased, the cytokine-mediated up-regulation of B7-H1 was more pronounced and caused an inhibition of allospecifc CD8^+^ T cell proliferation.

**Conclusion:**

Thus, IL-4 and TNFα, which could be released by immune cells of the tumor microenvironment, are able to control the B7-H1 expression in RCC thereby altering T cell responses. These data are of importance for understanding the complex interplay of tumor cells with immune cells orchestrated by a number of different soluble and membrane bound mediators and for the implementation of check point antibodies directed against B7-H1.

## Background

Renal cell carcinoma (RCC) is the most common kidney cancer type with an incidence of 5.8 in 100.000 people in the Western world [1] causing death of 5327 patients/year in Germany [2]. These results document the need for ongoing research to identify novel therapeutic strategies and to investigate mechanisms of tumor immune escape. RCC is considered an immunogenic tumor as demonstrated by a high frequency of tumor-infiltrating immune cells, a relatively high incidence of spontaneous recurrences as well as by the efficacy of immunotherapies, like DC-based vaccines, engineered autologous tumor cells, targeting T cell-tumor interaction, stem cell transplantation and treatment with cytokines [3].

B7 molecules are a growing protein family with diverse functions on both immune and tumor cells. They play in particular a key role in the crosstalk of the immune system and cancer tissues in different tumor entities [4]. B7 family members are mainly described to modulate T cell responses as second signal in cooperation with the first signal, the antigen recognition mediated by binding of the T cell receptor (TCR) with the major histocompatibility complex (MHC). These signals can be of co-inhibitory or co-stimulatory nature. Interestingly, also a reverse signaling in B7 family member expressing cells has been discovered [5]. The B7 family comprises B7-1 (CD80), B7-2 (CD86), B7-H1 (PD-L1), B7-DC (PD-L2), B7-H2 (ICOS-L), B7-H3, B7-H4 and B7-H6 [6,7]. Many tumors of distinct origin express B7-H molecules, in particular B7-H1. Therefore, monoclonal antibodies (mAb) targeting PDL1/B7-H1 on tumor cells or the PD1 receptor expressed by immune cells have been developed for the treatment of tumors. These antibodies are currently implemented in clinical trials demonstrating promising objective response rates in various tumors [8].

In the context of RCC B7-H1 expression of tumor lesions is associated with a worse prognosis of RCC patients [9]. The prognostic relevance of B7-H1 in RCC was further strengthened by the fact that B7-H1 mRNA is increased in early metastasis when compared to primary lesions suggesting that B7-H1 might serve as marker of a metastatic signature in RCC [10].

Cytokines are a family of modulatory proteins or glycoproteins that bind to their respective receptors on a variety of different immune and cancer cells thereby inducing different downstream signaling processes. Studies of cytokines are complicated due to their pleiotropy and apparent redundancies of action [11].

Over two decades one conventional treatment regime for patients with RCC included cytokines like IFN-α and IL-2. Despite the results were promising in preclinical settings [12-14], the clinical efficacy was rather poor with anti-tumoral responses ranging between 10 – 20% [15-18]. This might be due to the lack of knowledge of the tumor microenvironment, the molecular alterations and heterogeneity of tumors including those concerning the B7 family members in tumor and immune cells upon cytokine treatment.

With the exception of interferon (IFN)-γ [19,20] the effect of different cytokines on the regulation of B7-H molecules on RCC cells is widely unknown. Therefore, this study analyzed the regulation of B7-H molecules upon cytokine treatment in RCC in detail. B7-H1 surface expression was most dramatically altered upon IL-4 and TNFα. This enhancement occurred at the transcriptional level by direct upregulation of the B7-H1 promoter activity, which was associated with an inhibition of T cell proliferation.

## Materials and methods

### Cell lines and PBMC from healthy donors

The following RCC cell lines were used in the study and originally established from RCC patients in Mainz (MZ) or in Halle (Hal): MZ2514RC, MZ1257RC, MZ1790RC, MZ1774RC, MZ2733RC, MZ2877RC, Hal31RC, Hal162RC, Hal87RC and Hal149RC. The two melanoma cell lines BUF1088Mel and UKRV-Mel-14a have been recently described [21] and were either a kind gift from S. Ferrone (Pittsburgh, USA) or obtained from the European tumor cell line data base (ESTAB project; see http://www.ebi.ac.uk/ipd/estdab). Buffy coats were obtained from healthy donor (HD) of the blood bank of the University Hospital Halle. The Institutional Review Board (Ethics Committee) at the University Hospital in Halle (Germany) approved this study.

### Reagents

Monoclonal antibodies (mAb) for flow cytometric analysis were: αCD8, αHLA-I (clone B9.12.1), αCD40 and αICAM-1 from Beckman Coulter (Krefeld, Germany); αPD1, αCD80, αNF-κB (pS529), αIκB and αpSTAT6 from Becton Dickinson (Heidelberg, Germany); αB7-H4 from AbD serotec; αICOS, αB7-H4, αB7-H1, αB7-H2 and αCD107a from ebioscience (Frankfurt, Germany); αB7-H3, αTNFRI and fluorokine biotinylated human IL-4 staining kit were used from R&D systems (Wiesbaden, Germany). Respective isotypes were purchased from BD Bioscience or Beckman Coulter, respectively. The antibodies were used unconjugated and/or as direct conjugates with FITC, Alexa-488, PE, APC or PE-Cy7. Recombinant TNFα and IL-4 for the treatment of RCC cell lines were purchased from ImmunoTools (Friesoythe, Germany).

IL-2 (Proleukine, Pharmacy, University of Halle, Germany), phorbol myristate acetate (PMA), propidiumiodid and ionomycin from Sigma-Aldrich (Steinheim, Germany) were used. αCD8 microbeads were obtained from Miltenyi Biotech (Gladbach, Germany). RPMI1640 and DMEM were purchased from Invitrogen (Karlsruhe, Germany), X-VIVO15 from Lonza (Basel, Switzerland). The fix and perm kit for intracellular stainings was from BD Bioscience.

The following antibodies for cell culture were employed: αCD3 (clone OKT3) and αB7-H1 (clone MIHI) from ebioscience, purified mIgG1 and mIgG2a from Millipore (Eschborn, Germany) and αHLA-I (clone w6/32) obtained from culture supernatants of hybridomas.

### Flow cytometry

Flow cytometric analyses were essentially performed as recently described [21]. In brief, 1 × 10^5^ cells were stained with fluorescent-labeled antibodies, while dead cells were excluded using PI staining. For determination of the IL-4 receptor expression an indirect staining method using IL-4-biotin followed by Avidin-FITC according to manufacturers’ protocols was employed. For intracellular flow cytometric analyses of signal transduction components paraformaldehyde-fixed tumor cells were used, subsequently treated with permeabilizing buffer (methanol) prior to antibody staining. Flow cytometry was performed using either a FACSscan™, FACSCalibur™ or a FACSCanto™ (all Becton Dickinson) or FC500 (Beckman Coulter) flow cytometer and CellQuest™ or CXP™ and FlowJo™ (Tree Star) software, respectively.

### Cytokine treatment of tumor cells

3×10^5^ tumor cells/well were seeded into 6 well plates in DMEM/10% FCS. Cytokines (IL-4 at 1000U/ml and TNFα at 800 U/ml) were added the following day for 30 min or 4–72 hrs as indicated for the subsequent analysis.

### PCR analysis

Total cellular RNA from frozen cell pellets was extracted using RNAeasy MiniKit (Qiagen Hilden, Germany) and reversely transcribed into cDNA (Fermentas, St. Leon-Rot Germany) as recently described [22]. Semi-quantitative RT-PCR from cellular RNA was performed using the following oligonucleotide primers: For IL-4 *fw: 3′ cagttctacagccaccatgaga 5′ rev: 3′ catgatcgtctttagcctttc 5′* for, IL-4Rα *fw: 3′ tctacttgcgagtggaagatga 5′ rev: 3′ ctccaaatgttgactgcatagg 5′,* TNFα *fw: 3′ gtgcttgttcctcagcctct 5′ rev: 3′ gcttgtcactcggggttc 5′,* TNFRI *fw: 3′ gccaggagaaacagaacacc 5′ rev: 3′ gggataaaaggcaaagacca 5′* and for β-actin *fw: 3′ tcctgtggcatccacgaaact 5′ rev: 3′ gaagcatttgcggtggacgat 5′.* Realtime PCR (Cybr Green, Invitrogen) analysis for B7-H1 and B7-H4 from cellular RNA was performed using the following oligonucleotide primers: H1: fw: 3′ *gaactacctctggcacatcct* 5′ rev: 3′ *gcccattccttcctcttgtc* 5′, H4: fw: 3′ aggcttctctgtgtgtctcttc 5′ rev: 3′ cttgctcttgtttgctcactcc 5′.

### Cloning of the reporter gene vector

Genomic DNA was isolated from the B7-H1 expressing melanoma cell line UKRV-Mel-14a using the QIAamp DNA Mini Kit (Qiagen) according the manufacturers’ protocol. The B7-H1 promoter was amplified by PCR with Taq DNA polymerase Kit (Invitrogen) employing the forward primer 5′-AAAGGTACCTAGAAGTTCAGCGCGGGATA-3′ and the reverse primer 5′-AAAGGATCCCAGCGAGCTAGCCAGAGATA-3′. The specific PCR product was purified and cloned into the pMiR REPORT vector (Ambion, Austin, Texas, USA) using the restriction enzymes KpnI and BamHI (Fermentas) replacing the CMV promoter as recently described [23]. For replacing the luciferase (luc) reporter gene by the red fluorescent m-cherry protein, the m-cherry sequence was amplified from the pmR-m-cherry vector (Clontech, Mountain View, CA, USA) applying the forward primer 5′-AAAGGATCCATGGTGAGCAAGGGCGAGGA-3′ and the reverse primer 5′-AATGTGGTATGGCTGATTAT-3′. The PCR product was digested with BamHI (Fermentas) and SpeI (NEB, Ipswich, MA, USA) and cloned behind the B7-H1 promoter sequence in the pMiR REPORT backbone replacing the luciferase gene. The plasmid map is shown in Additional file 1: Figure S1.

### Cell transfection

The reporter gene plasmid was stably transfected into the melanoma cell line BUF1088Mel using the Effectene Transfection Reagent (Qiagen, Hilden, Germany). Stable transfectants were selected with puromycin (pur) and a pur-resistant batch culture was generated. Transfected cells were cytokine treated as described above and flow cytometric analyses were performed 72 hrs post stimulation.

### Tumor-T cell co-culture assays

Tumor cells were pretreated with cytokines as described above, detached, washed with PBS (3 x times), counted and seeded with 1 – 2 × 10^5^ into 96 or 24 well plates. Peripheral blood mononuclear cells (PBMC) were obtained by Ficoll gradient from buffy coats of healthy volunteers. T cells were sorted for CD8^+^ cells (purity > 98%) and co-cultivated tumor cells as described [21]. For proliferation assays, T cells were labeled with CDFA-SE (Lifetechnologies, Darmstadt, Germany) according to manufacturers’ instructions) and tumor cells were pretreated with αHLA-I or anti-B7-H1 for 30 min prior to 5 day co-culture assays. Proliferation data are presented as division index (DI) that is the average number of cell divisions that a cell in the original population has undergone. For the determination of IFNγ secretion tumor cells were co-cultured with T cells for 4 hrs. Cell culture medium for the coculture assays was X-VIVO15.

### Detection of cytokine release

To determine IFN-γ secretion of T cells the IFN-γ secretion assay (Miltenyi) was performed following the manufacturer’s instructions. T cells stimulated with PMA/ionomycin (10 ng/ml and 1 μg/ml) served as a positive control. TNFα production of tumor cells was analyzed from culture supernatants using a TNFα-specific ELISA according to manufacturer’s instructions (ebioscience).

### Statistical analysis

Statistical analyses were performed using Prism 3.0 and depending on controlled data normality distribution Mann Whitney U Test or student’s t test was used.

## Results

### Cytokine receptor expression as a prerequisite for cytokine activity in RCC cells

**Figure 1:**
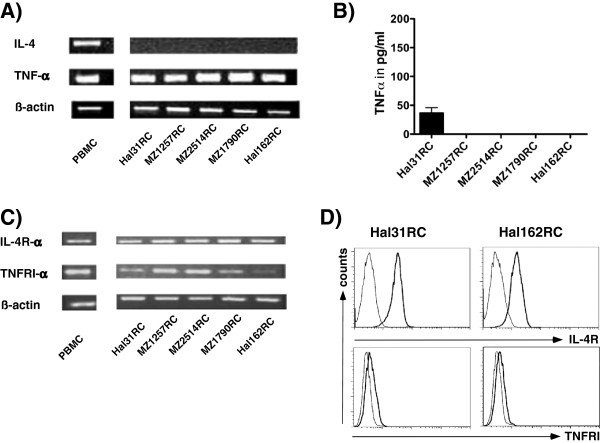
**Expression analysis of IL-4, TNFα and their receptors in RCC cell lines. A)** Constitutive TNFα mRNA expression, but lack of IL-4 mRNA expression was determined by conventional qPCR in different RCC cell lines (Hal31RC, MZ1257RC, MZ2514RC, MZ1790RC, Hal161RC) and in PBMC, which served as positive control. Representative data of at least 3 different experiments are shown. **B)** TNFα secretion of different RCC cell lines TNFα secretion was determined in Hal31RC, MZ1257RC, MZ2514RC, MZ1790RC, Hal161RC using ELISA. Representative data out of at least 3 different experiments are shown. **C)** The IL-4Rα and TNFRI-α expression was determined by qPCR in the different RCC cell lines and in PBMC, which served as positive control. Representative data of at least 3 different experiments are shown. **D)** Flow cytometric analyses of IL-4R and TNFαRI demonstrated the constitutive expression of IL-4R and TNFRI on Hal31RC and Hal161RC. The results are expressed as histograms. Bold line: staining, thin line: control. TNFRI is stained with an anti-TNFRI-PE labeled antibody, bold line: staining, thin line: isotype control. Representative data of at least 3 different experiments are shown.

The constitutive expression of the IL-4 and TNFα receptors and and their ligands were determined in RCC cell lines. Using conventional qPCR TNFα, but not IL-4 mRNA expression levels were detected in the different RCC cell lines (Figure 1A). Despite prominent TNFα transcription rather low secretion levels of this cytokine were detected in one/five RCC cell lines tested (Figure 1B). In contrast, both the IL-4Rα and TNFRI were expressed at the mRNA (Figure 1C) and protein level (Figure 1D) as determined by qPCR and flow cytometry, respectively.

### IL-4 and TNFα synergistically enhance B7-H1 expression in RCC cells

**Figure 2:**
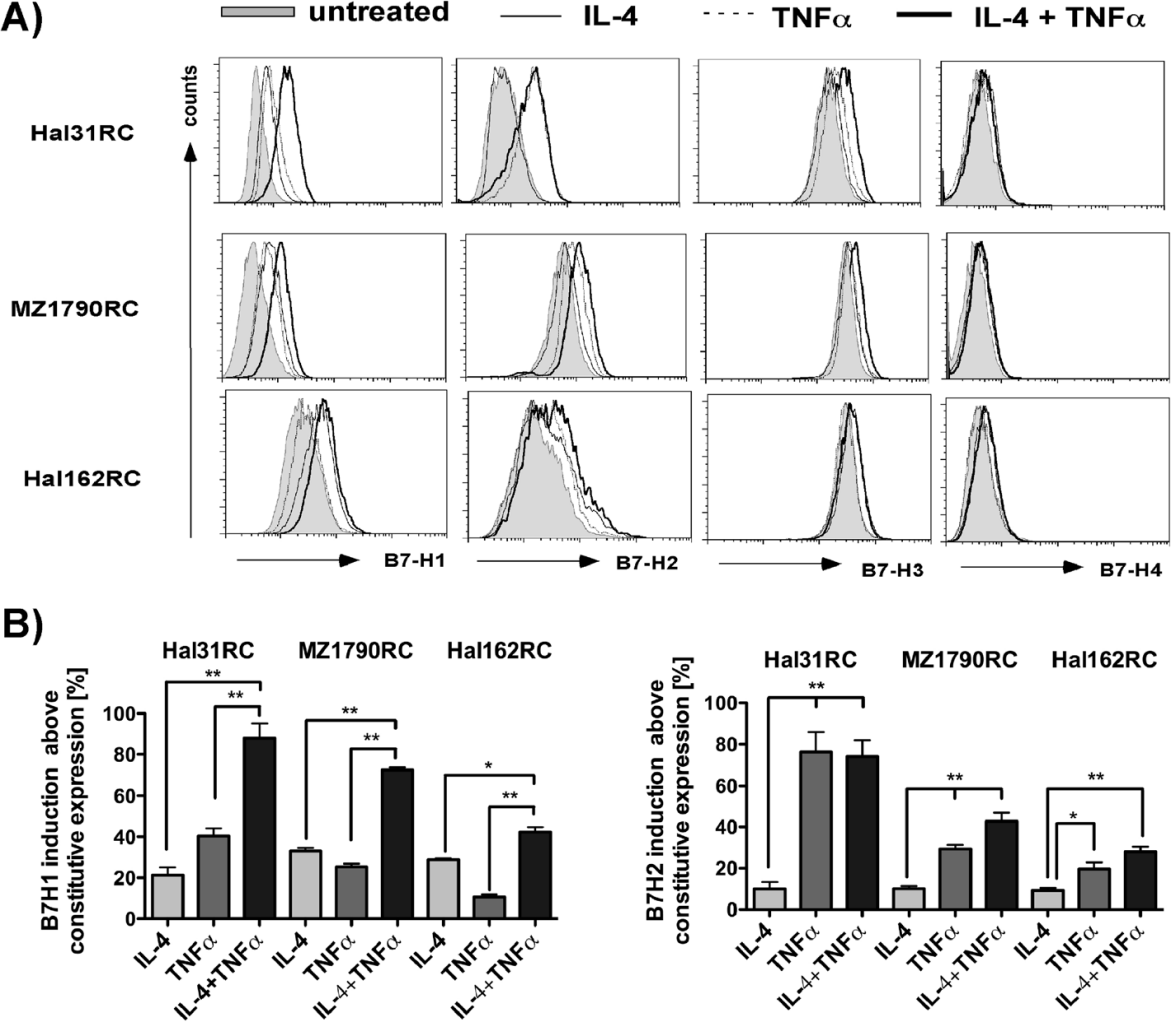
**Significant increase of B7-H1 surface expression by IL-4 and TNF**α **treatment of RCC cell lines.** Different RCC cell lines (Hal31RC, Hal161RC, MZ1790RC) were treated with either IL-4 or TNFα alone before B7-H expression was determined by flow cytometry as described in Materials and Methods at 72 hrs. **A)** Shown are FACS histogram overlay plots for B7-H1, B7-H2, B7-H3 and B7-H4. Each plot shows overlay graphs, depicting constitutive protein expression and expression upon single or combined cytokine treatment. Representative data of at least 3 different experiments are shown. **B)** Cytokine-mediated induction of B7-H1 and B7-H2 in % using constitutive expression levels as reference value. Combined data of 3 different experiments are given.

**Figure 3:**
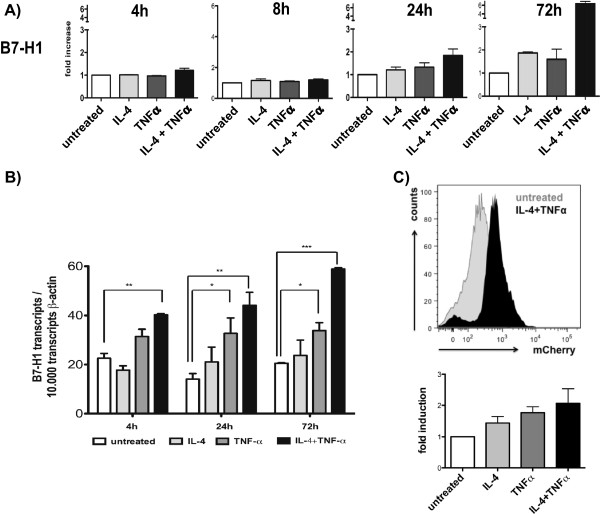
**Time kinetic of B7-H1 expression and B7-H1 promoter targeting upon combined cytokine treatment with IL-4 and TNFα. A)** Hal31RC was treated with either IL-4 or TNFα alone and with the combination of the two cytokines. B7-H1 protein expression was analyzed at the time points indicated by flow cytometry. Shown are fold increase in B7-H1 expression compared to untreated cells. Representative data of at least 3 different experiments are shown. **B)** B7-H1 mRNA expression in Hal31RC upon treatment with either IL-4 or TNFα alone and with the combination of the two cytokines for the time points indicated are shown. Using a B7-H1 plasmid as standard, B7-H1 transcript levels per 10.000 transcripts β-actin are shown. Data are obtained by real time PCR. Representative data of 3 different experiments are shown. **C)** BUF1088Mel was transfected with the mCherry B7-H1 promoter construct and stimulated with IL-4 and TNFα. The mCherry fluorescence was measured after 72 hrs by FACS and a representative histogram plot is depicted. The bar graph shows the combined result of a total of 3 independent assays.

A heterogeneous, but constitutive cell surface protein expression of B7-H family members was detected in the tested RCC cell lines (Table 1). While B7-H1 to B7-H4 were expressed on all RCC cell lines, the degree of expression was quite diverse with the highest levels for B7-H3 followed by B7-H2 and B7-H1, whereas only weak, but detectable B7-H4 expression levels were found (Table 1). In addition, qPCR analysis revealed low B7-H4 mRNA levels (Additional file 2: Figure S2). 2/3 RCC lines were also weakly positive for B7-DC (data not shown). Upon treatment with either IL-4 and TNFα alone or in combination, the B7-H molecule expression was differentially affected: B7-H4 transcription (data not shown) and protein expression (Figure 2) was unaltered by this cytokine treatment. Despite high constitutive expression levels, a slight cytokine-mediated induction of B7-H3 was shown, which was most prominent in RCC31 upon IL-4 and TNFα treatment. B7-H2 was induced by TNFα, but not by IL-4 in the RCC cell line analyses, while combined TNFα with IL-4 treatment had no additional effects (Figure 2). The most prominent induction was found for B7-H1 in all RCC cell lines by treatment with either cytokine, while combination treatment caused synergistic effects on B7-H1 protein expression (Figure 2 and Figure 3A), with a < 6 fold increase in RCC31 cells (Figure 3A). Furthermore, IFNγ treatment of RCC cells increased B7-H1 mRNA and protein expression (data not shown) confirming recent reports [19].

**Table 1:** Constitutive B7-H1-B7-H4 expression in renal cell carcinoma cell lines as determined by FACS, isotype control stainings were implemented

**cell lines**	**B7-H1**	**B7-H2**	**B7-H3**	**B7-H4**
**Hal31RC**	+	+	++	+/-
**Hal78RC**	+	+	++	+/-
**Hal149RC**	+	+	++	+/-
**Hal162RC**	++	++	++	+/-
**MZ1774RC**	+	+	+++	+/-
**MZ1790RC**	+/-	++	++	+/-
**MZ1257RC**	+	+	++	+/-
**MZ2514RC**	+	+	++	+/-
**MZ2733RC**	+	+	++	+/-
**MZ2877RC**	+	+	++	+/-

+/- : 5-20% positive; +: 20-50% positive; ++ : > 50-100% positive; +++: 100% positive, and staining intensity > 10 fold to isoytpe control.

In addition, other cytokines like IFNα, IFNβ, TGFβ, IL-6 and IL-10 were tested for their ability to modulate B7-H expression on RCC cells, but none of them altered the expression levels of these molecules. The lack of the cytokine-mediated effects on B7-H family members was specific, since these cytokines modulate e.g. HLA class I molecule expression (data not shown).

### Transcriptional control of cytokine-mediated induction of B7-H1

To identify the kinetics of IL-4- and TNFα-mediated regulation of B7-H1 expression RCC cells were treated for different time points with IL-4 and TNFα alone or in combination before qPCR and flow cytometry was performed. Already 4 hrs post combined cytokine treatment a significant induction of B7-H1 mRNA was detected (Figure 3B). Treatment with IL-4 alone only marginally increased the B7-H1 transcript levels throughout the time points tested, whereas TNFα alone induced a significant increase in B7-H1 mRNA levels at 24 hrs post treatment. Combined cytokine treatment had always the strongest impact on B7-H1 mRNA (Figure 3B). An enhanced protein expression was only detected at later time points, with a maximal induction at 72 hrs post cytokine treatment. These results pointed towards a regulation at the transcriptional rather than posttranscriptional level, e.g., by protein stabilization.

In order to test B7-H1 regulation at the transcriptional level in more detail, promoter binding prediction program (TESS) was used to determine direct binding sites for STAT6 (downstream of IL-4 signaling) and NFκB (p65, downstream of TNFα) on the B7-H1 promoter (-952 to -1 bp before transcript start). A weak binding site for STAT6 (TTACAAGAA) and two overlapping high affinity binding sites for NFκB (GGAAAGTCCA; AGGAAAGTCCAAC) were found in the B7-H1 promoter. One NFκB binding site in the B7-H1 promoter has been shown to control B7-H1 expression in renal tubular cells [24].

Based on this finding B7-H1 promoter studies were performed [25]. The wild type B7-H1 promoter construct hooked to the mCherry fluorescent protein was stably transfected into BUF088Mel cells (Additional file 1: Figure S1). Transfectants were subsequently left untreated or treated with either cytokine (IL-4 or TNFα) alone or in combination before promoter activity was determined 72 hrs later. As shown in Figure 3C, an enhanced B7-H1 promoter activity as determined by flow cytometry of the mCherry fluorescence was found upon single and more pronounced upon combined treatment with IL-4 and TNFα. This suggests a transcriptional upregulation of B7-H1, which might be mediated by activation of STAT6 and NFκB.

### Alterations in pSTAT6, NFκB, LFA-1, CD40 and HLA class I antigen expression levels upon treatment of RCC cells with IL-4 and TNFα

In addition to the regulation of B7-H molecules by cytokine treatment downstream signal cascade components and other processes known to be of relevance for the T cell/tumor interaction and the growth behavior of RCC cells were investigated.

A growth inhibition of RCC cells was detected upon IL-4 addition, which was not further pronounced in the presence of TNFα (Additional file 3: Figure S3). In contrast, TNFα alone had no influence on the cell proliferation, which is in discrepancy to earlier published data showing an increased proliferation upon TNFα treatment, but a strong influence of the culture media conditions were found [26].

**Figure 4:**
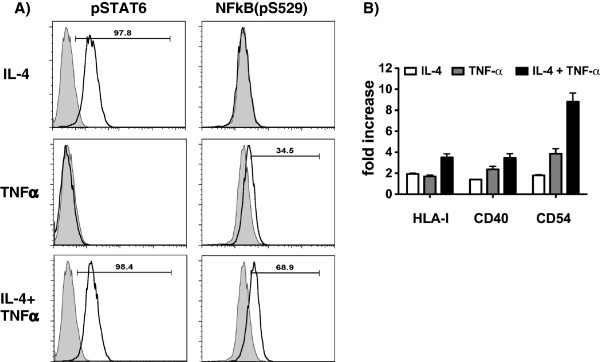
**Involvement of signaling components and change of HLA-I, CD40 and CD54 expression upon combined cytokine treatment with IL-4 and TNFα in RCC cell lines.** Hal31RC was treated with either IL-4 or TNFα alone and with the combination of the two cytokines. **A)** Intracellular pSTAT6 as well as NFκB (pS529) at 30 min post stimulation were analyzed by flow cytometry. Gray filled graph represents constitutive expression and black bold line represents cytokine-induced expression of either signaling component. Numbers on gates are percentage of positive cells compared to medium control. Representative data of at least 5 different experiments are shown. **B)** HLA class I, CD40, CD54 expression 72 hrs post cytokine treatment were analyzed by flow cytometry. Data are presented as fold increase when compared to constitutive expression of these molecules on untreated Hal31RC. Data are mean ± SEM of 9 different experiments.

Following IL-4 treatment alone or in combination with TNFα phosphorylation of STAT6, a key signal molecule downstream the IL-4 receptor, was detected by flow cytometry (Figure 4A). A known key downstream effector of TNFα signaling is represented by NFκB. An enhanced expression and activation of NFκB was found in TNFα as well as TNFα- and IL-4-treated RCC cells (Figure 4A). Next to the upregulation of NFκB the inhibitory subunit of NFκB, named IkB, was downregulated upon TNFα treatment in RCC cells (data not shown). Downregulation/degradation of IκB as a part of the classical NFκB pathway activation has been described earlier [27].Additional important molecules for T cell/tumor communication are CD40, a costimulator of the TNF-R family, the adhesion molecule CD54 (LFA-1) and HLA class I antigens. As shown in Figure 4B all these surface molecules were upregulated by the treatment of RCC cells with IL-4 and TNFα, either alone or in combination. The combined treatment of IL-4 and TNFα had an additive (CD40, HLA class I) or a synergistic effect (LFA-1) on the protein expression, respectively (Figure 4B).

### B7-H1 blocks allospecific T cell proliferation

**Figure 5:**
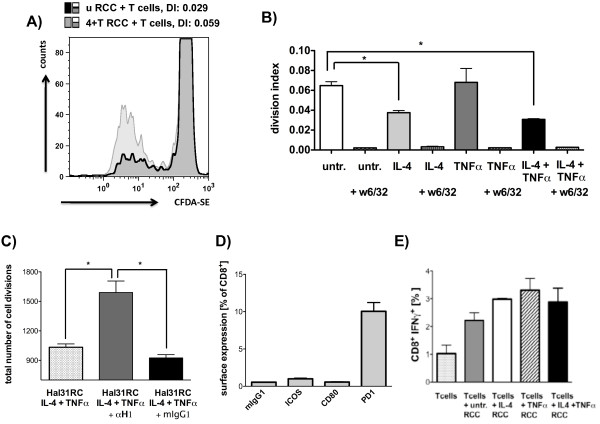
**Block of allogeneic CD8**^**+ **^**T cell proliferation upon coculture with RCC cells pretreated with IL-4 and TNFα. A-C)** Hal31RC cells were treated with either IL-4 or TNFα alone and with the combination of both cytokines. CD8^+^ T cells from allogeneic PBMCs were sorted, CFDA-SE labeled and cocultured with cytokine pretreated RCC31Hal. **A)** Representative FACS histogramm plots of proliferated CD8^+^ Tcells cocultured with untreated (u RCC) as compared to IL-4 or TNFα pretreated Hal31RC (4 + T RCC) are shown. The division index (DI) is given. **B)** Summary of CD8^+^ T cell proliferation on cytokine pretreated Hal31RC. The division index (DI) is given. Pan anti-HLA class I antibody (w6/32) served as specificity control. Data are means of 4 different donors ± SEM. **C)** Partial reversal of T cell proliferation inhibition upon coculture of CD8^+^ T cells with anti-B7-H1 antibody or respective isotype control preincubated Hal31RC. Hal31RC were pretreated with IL-4 and TNFα for 3 days. Data represent the mean of 3 different donors ± SEM. **D)** Analyses of constitutive B7H ligand expression, ICOS, CD80 and PD-1 on allogeneic CD8^+^ T cells prior to coculture with RCC31. Data represent means of 7 different donors ± SEM. **E)** IFNγ production of isolated CD8^+^ T cells upon coculture of cytokine pretreated Hal31RC cells. Data are means of 3 different donors ± SEM.

B7-H1 has been shown to modulate T cell responses. [19,28] To test the functional consequences of increased B7-H1 on RCC cells upon IL-4 and TNFα treatment co-culture assays of tumor cells with allospecific T cells were performed. A decreased CD8^+^ T cell proliferation was detected upon co-cultivation of T cells with IL-4 pretreated as well as with IL-4 and TNFα pretreated tumor cells (Figure 5A and B). The direct influence of both cytokines on T cells was eliminated by 3 times washing of cytokine-treated tumor cells, prior to co-culture assays. The decreased proliferation of CD8^+^ T cells upon co-culture with IL-4- and TNFα-pretreated tumor cells could be partially converted by the use of an anti-B7-H1 blocking antibody as proof for the inhibitory role of B7-H1 on tumor cells (Figure 5C).

To identify the possible binding partner of B7-H1 on T cells, an anti-PD1 antibody was used for staining to detect the most likely candidate PD1. Indeed, PD1 was expressed in 10.05 ± 1.17% of CD8^+^ T cells analyzed (Figure 5D). Since CD80 expression on T cells has been postulated [29] and CD80 can additionally bind B7-H1 [30] its CD80 expression was determined on T cells. In addition, since B7-H2 expression is known to be increased upon treatment with TNFα, ICOS, the ligand of B7-H2 was analyzed on CD8^+^ T cells prior to co-culture assays. As shown in Figure 5D, CD80 and ICOS were only very weakly expressed on CD8^+^ T cells.

Important CD8^+^ T cell effector functions are cytokine production and the ability to kill target cells. As determined flow cytometry IFNγ secretion by CD8^+^ T cells was unaltered upon co-culture with cytokine-treated RCC cells (Figure 5E). Furthermore, the killing ability of CD8^+^ T cells was determined by measurement of CD107a degranulation, CD8^+^ T cells upregulated CD107a expression upon co-culture with RCC cells, but no specific changes with respect to co-culture with cytokine pretreated tumor cells were found (data not shown).

## Discussion

The present study was undertaken to discover the distribution and regulation of B7-H family members in RCC by cytokines released from immune cells of the tumor microenvironment. Interesting, a synergistic increase of B7-H1 surface expression in RCC cells upon treatment with IL-4 and TNFα was found. As early as 4 hrs after treatment, B7-H1 mRNA was significantly enhanced resulting in 6- fold increase in protein surface expression by 72 hrs and was mediated by an upregulation of the B7-H1 promoter activity by combined IL-4 and TNFα treatment. In addition, the increase in B7-H1 protein surface expression on RCC cells was associated with a decreased allospecific T cell proliferation upon co-culture experiments.

Detailed analysis showed a constitutive, but variable surface expression of B7-H1 to B7-H4 molecules on RCC cells. Expression of B7-H1, B7-H3 and B7-H4 *in situ* has been correlated with a worse clinical outcome of RCC patients [9,31,32]. In all tested RCC cell lines, B7-H4 was rather weakly expressed. In contrast B7-H3 was strongly expressed on RCC cell lines. To the best of our knowledge, we are the first to show B7-H2 expression on RCC cells. There exist random reports showing expression of this molecule in human tumors such as glioblastoma [33] and melanoma [34]. B7-H2 on glioma cells leads to an increase in T cell-mediated anti-tumor immunity [35].

In addition, B7-H1 expression on RCC cells was confirmed with a weak or intermediate expression level by all RCC cell lines tested. Testing of the constitutive cytokine expression revealed a weak TNFα production by one RCC cell line, while all others were negative. These data are in line with earlier reports describing that some RCC cells are able to produce TNFα [36]. Furthermore, RCC cells in our study lack IL-4 expression thereby confirming previous published data [37]. A prerequisite to respond to a particular cytokines is the expression of the respective cytokine receptors. Both the IL-4R as well as TNFRI expression was found on all RCC cells tested. TNFα acts via TNFRI and II both expressed on RCC cells [38]. IL-4R expression on RCC cells *in vitro* and *in situ* has been demonstrated before [39]. Interestingly, structural differences for IL-4R on RCC cells when compared to immune cells exist, which might partially explain the differential outcome of IL-4 action in these cells [37]. Most of the RCC cell lines tested in the present study showed a very good and reliable response to IL-4 and TNFα treatment as demonstrated by phosphorylation of STAT6, enhancement of pNFκB and downregulation of IkB.

Given the importance of B7-H molecules for the outcome of RCC patients and the presence of cytokines in the tumor microenvironment, the regulation of these family members upon treatment with various cytokines was determined. As already described, upregulation of B7-H1 expression by IFNγ was confirmed [19]. In addition the most prominent effect on the regulation of B7-H1 was found using combined IL-4 and TNFα treatment. B7-H1 was transcriptionally controlled by these cytokines. B7-H2 was only upregulated by TNFα treatment, but not by IL-4.

IL-4 and TNFα can both be produced by different immune cells and thus represent components of the tumor microenvironment. In RCC TNFα is produced by tumor-associated macrophages (TAM) [40]. TAMs can be subdivided into classical M1 phenotype macrophages that produce and dependent on proinflammatory cytokines, such as TNFα and into alternative M2 phenotype macrophages [41]. M2 macrophages are characterized by the production of IL-10, TGF-β and are induced by IL-4. The ratio of M1/M2 TAMs together with the number and phenotype of dendritic cells, myeloid derived suppressor cells (MDSC) and the Th1/Th2 balance determine the cytokine milieu and thereby the anti-tumor response in the tumor microenvironment. Already 15 years ago CD4^+^ Th cells of the Th1 (predominantly IFNγ) and Th2 (predominantly IL-4) cells as well as CD8^+^ T cells have been shown to play a key role for an effective anti-tumor response. Furthermore, IL-4 has a tremendous impact on the anti-tumor immunity by shifting the Th1/Th2 balance [42]. The importance for IL-4 in RCC is demonstrated by the existence of a functional polymorphism in the IL-4 gene (-590 T) leading to an enhanced expression of this cytokine, which is correlated with an increased risk of developing RCC [43] and a decreased survival [44] when compared to RCC patients carrying the other haplotype (-590C). In contrast to earlier reports, IL-4 can reduce tumor growth suggesting that the time point and local distribution of high IL-4 levels have an impact on RCC progression. Of importance, microarray data reveal a positive correlation of B7-H1 with TNFα, NFkB and STAT6 (http://r2.amc.nl) in kidney tumor tissue in vivo, nicely supporting our data of a linked B7-H1 expression with these cytokines.

In addition to IFN-γ, the regulation of B7-H expression by cytokines has been studied earlier, but mostly on immune cells and not on RCC cells. Kryzek and co-authors showed an increase of B7-H4 in TAMs upon treatment with IL-6 or IL-10 [45]. Similar to our findings TNFα upregulates B7-H2 on embryonic fibroblasts [46] and endothelial cells [47], while IL-4 did not modulate B7-H2 and the combined treatment had no additional effect [47]. Interestingly, on human endothelial cells TNFα together with IFNγ synergistically affect induction, whereas TNFα alone did not induce any B7-H1 expression [48].

As already proposed by and confirmed in this study combined IL-4 and TNFα treatment exerts a synergistic effect on the increase of HLA class I antigen expression in RCC cells, which might enhance T cell-based anti-tumor responses [13]. This was further supported by an increased expression of components of the APM leading to increased HLA class I surface antigen expression in RCC cells upon IFN-γ treatment [30]. Since another hallmark of effective T cell response is cell adhesion, ICAM1 expression was analyzed. ICAM1 expression was highly upregulated upon combined IL-4 and TNFα treatment, which can support T cell/tumor interaction. This is in line with published data demonstrating that TNFα induces ICAM1 expression [49].

As functional consequences of cytokine-mediated enhanced B7-H1 on RCC tumor cells, a decreased allospecific CD8^+^ T cell proliferation was found, which could be partially converted by addition of an anti-B7-H1 antibody. Since PD1 was the only substantially expressed receptor on the CD8^+^ T cells used in the co-culture assays, it is therefore most likely responsible for this effect. This assay nicely resembles the *in vivo* situation, since enhanced PD1 expression on tumor-infiltrating immune cells has been found in RCC and could be associated with poor patients’ prognosis [50]. However, IFN-γ and CD107a mobilization was not altered in CD8^+^ T cells upon co-culture with cytokine pre-treated tumor cells. An inhibitory effect of B7-H1 on CD8^+^ T cells has already been described in different studies. A block in T cell proliferation owing to B7-H1 has been shown before with overexpression or blocking of B7-H1, but not with cytokine-pretreated cells [51,52,20]. Additionally, a direct decrease of CD8^+^ T cell killing and cytokine production upon co-culture assays with anti-B7-H1 blocking antibody for human TCR tg CD8^+^ T cells in RCC has been found [19]. On the other hand, Dong and coworkers (2002) showed that TCR tg human CTL are equally able to kill B7-H1 over-expressing melanoma cells, but a B7-H1 dependent induction of T cell apoptosis was detected [28]. Together with the findings of this study the data demonstrate the powerful influence of B7-H1 on the modulation of different T cell effector responses, which highly appear to depend on the co-culture systems chosen.

The observed block of T cell proliferation on CD8^+^ T cells co-cultured with single IL-4-treated RCC cells could be due to an additionally interaction of PD1 on T cells with its second ligand B7-DC. A weak constitutive expression of B7-DC was found that could be also a subject to regulation by IL-4 similar as shown for macrophages [53] possibly not to TNFα as analyzed for monocytes [52]. B7-DC has been shown to inhibit human T cell proliferation by PD1 binding [51].

High dose TNFα has been used to treat solid tumors, but due to many side effects upon systemic administration the success rate has been rather low, and strategies to administer this cytokine more locally had been developed for e.g. melanoma patients [54], but have not been used to treat RCC.

Phase II clinical trials using IL-4 for the treatment of RCC were not beneficial for these patients [16,18]. This might be at least partially explained by the decreased T cell proliferation capacity upon co-culture assays with IL-4-treated RCC cells. TNFα alone had no effect on T cell effector responses in the setting used in our study, although TNFα treatment exerted beneficial anti-tumor effects in a xenograft mouse model with RCC tumors [12].

## Conclusion

Concluding the data, this study showed for the first time a detailed analysis of B7-H molecule regulation upon cytokine treatment in RCC. B7-H1 exhibited the strongest sensitivity to IL-4 and TNFα by a synergistic upregulation in RCC cells and is controlled at the transcriptional level by direct promoter targeting. The importance of this B7-H1 induction is demonstrated by an inhibition of T cell proliferation thereby contributing to the proposed significance of B7-H1 in cancer immunity. The study furthermore supports the rational of using B7-H1/PD1 checkpoint antibodies for the treatment of tumor patients.

## Abbreviations

APM: Antigen processing machinery; CTL: Cytotoxic T lymphocyte; NFκB: Nuclear factor kappa B; IFN: Interferon; IL: Interleukin; luc: Luciferase; mAb: Monoclonal antibodies; MHC: Major histocompatible complex; RCC: Renal cell carcinoma; STAT: Signal transducer and activator of transcription; TAA: Tumor-associated antigens; TAM: Tumor-associated macrophages; TCR: T cell receptor; TNF: Tumor necrosis factor.

## Competing interests

The authors declare that they have no competing interests.

## Authors’ contributions

DQ carried out main part of experiments, interpretation of results and wrote the manuscript, SJ-B carried out the promoter studies, UM performed part of the FACS analysis and the real time PCR experiments, BSc performed part of the T cell assays and BSe conceived of the study, participated in its design and coordination and was involved in writing the manuscript. All authors read and approved the final manuscript.

## Supplementary Material

Additional file 1: Figure S1 Reporter gene plasmid map for B7-H1. Click here for file

Additional file 2: Figure S2 B7-H4 mRNA as determined by real time PCR is given. Transcript numbers are calculated using a β-actin and B7-H4 plasmid as template. Representative data from 2 experiments are shown. Click here for file

Additional file 3: Figure S3 Growth inhibition of cells treated with IL-4 and the combination of IL-4+TNFα for 3 different RCC cell lines is shown. Same numbers of cells were seeded into wells and cells were equally detached and counted using trypan blue exclusion at the end of culture time (72hrs). Combined data of three different experiments are shown. Click here for file
