# Supplementary figures and images for: Synergistic effects of IL-4 and TNFα on the induction of B7-H1 in renal cell carcinoma cells inhibiting allogeneic T cell proliferation
Source: J Transl Med. 2014 May 30;12:151. doi: 10.1186/1479-5876-12-151 (PMC4079621; doi:10.1186/1479-5876-12-151)

## Slide 1
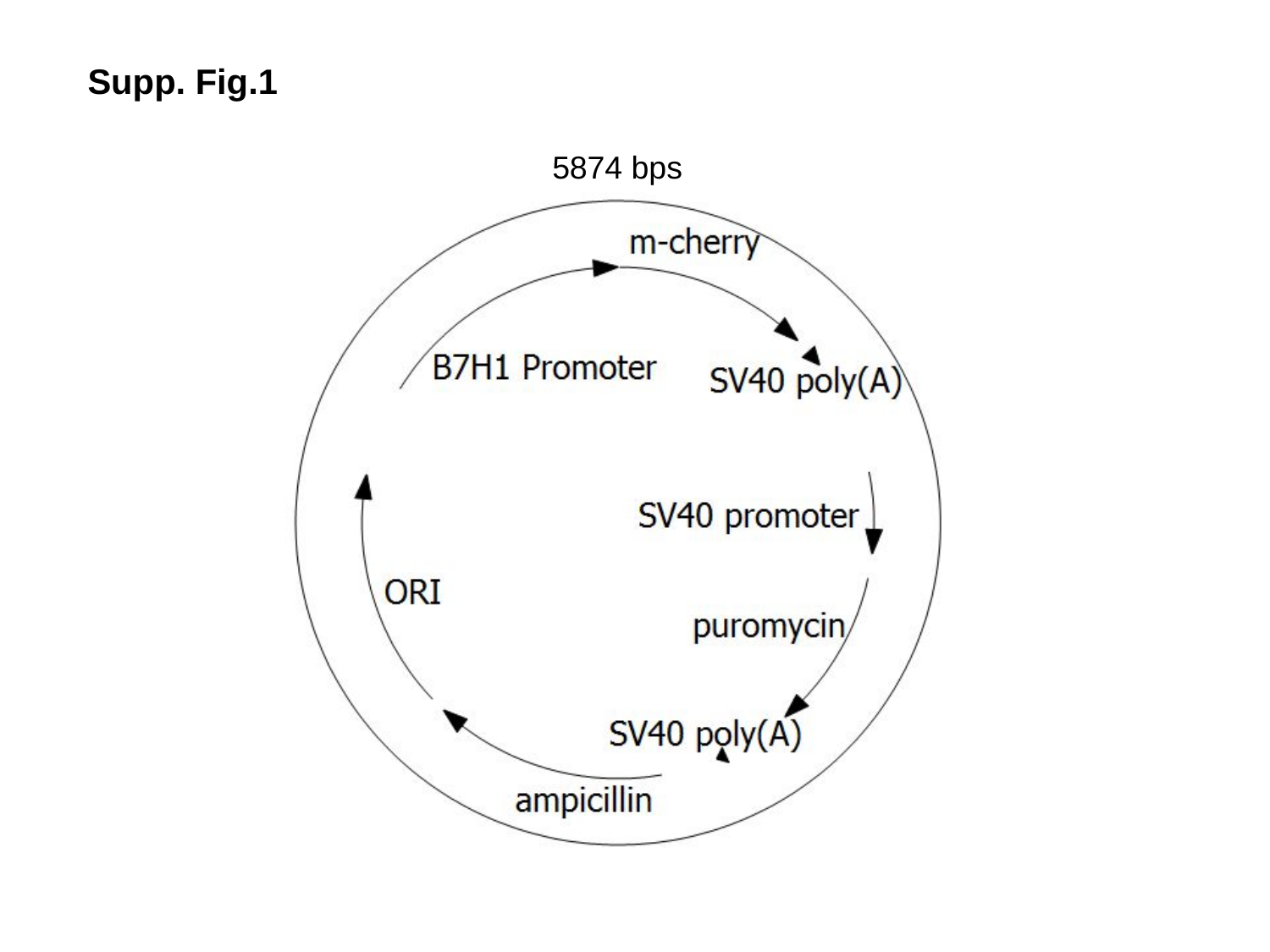

Supp. Fig.1
5874 bps

Supplement: Additional file 1: Figure S1 — Reporter gene plasmid map for B7-H1. [file 1479-5876-12-151-S1.ppt]
