# Supplementary material for: Synergistic effects of IL-4 and TNFα on the induction of B7-H1 in renal cell carcinoma cells inhibiting allogeneic T cell proliferation
Source: J Transl Med. 2014 May 30;12:151. doi: 10.1186/1479-5876-12-151 (PMC4079621; doi:10.1186/1479-5876-12-151)
Supplement: Additional file 2: Figure S2 — B7-H4 mRNA as determined by real time PCR is given. Transcript numbers are calculated using a β-actin and B7-H4 plasmid as template. Representative data from 2 experiments are shown. [file 1479-5876-12-151-S2.pptx]

## Slide 1
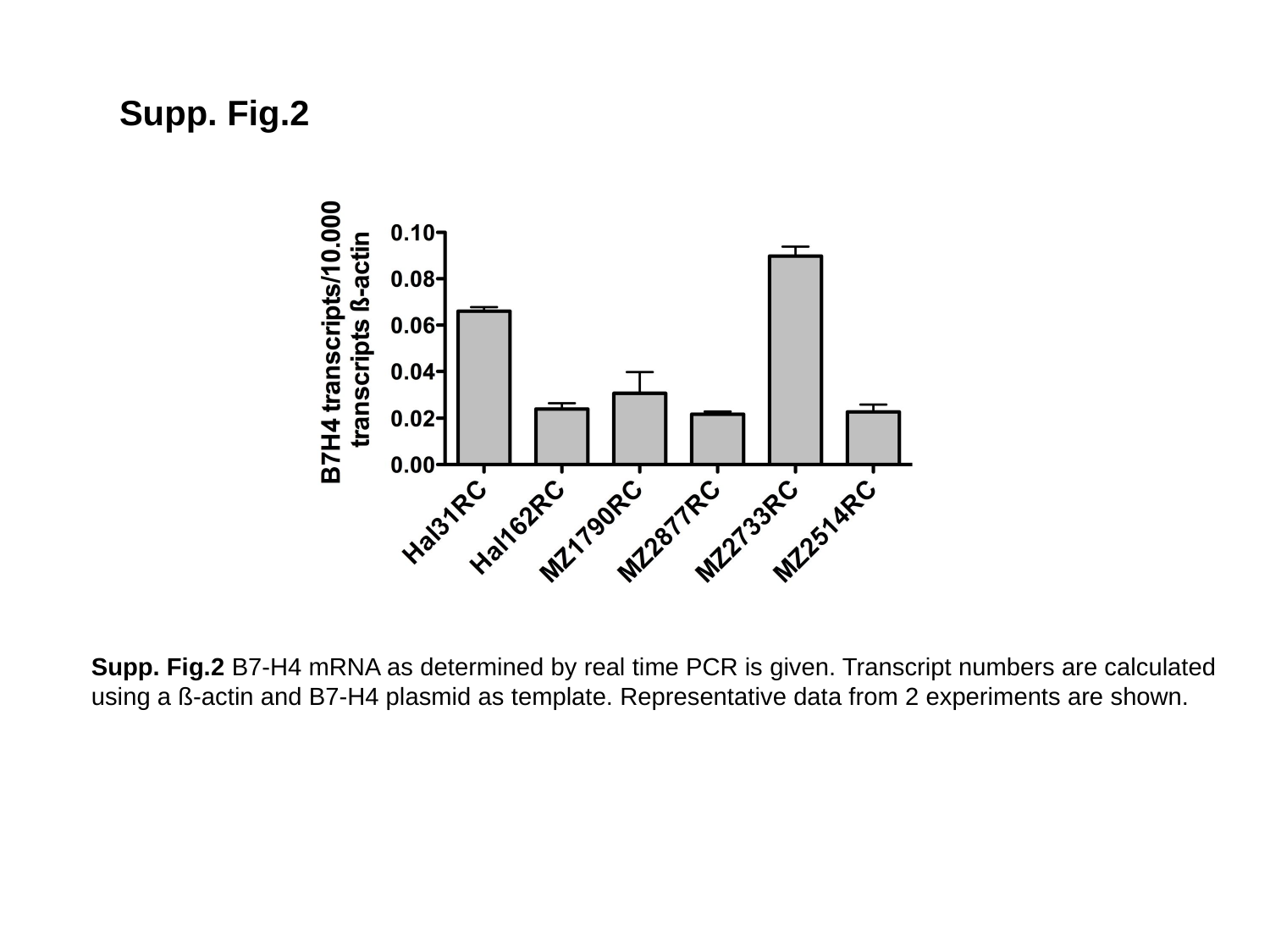

Supp. Fig.2
Supp. Fig.2 B7-H4 mRNA as determined by real time PCR is given. Transcript numbers are calculated
using a ß-actin and B7-H4 plasmid as template. Representative data from 2 experiments are shown.
