# Supplementary material for: Synergistic effects of IL-4 and TNFα on the induction of B7-H1 in renal cell carcinoma cells inhibiting allogeneic T cell proliferation
Source: J Transl Med. 2014 May 30;12:151. doi: 10.1186/1479-5876-12-151 (PMC4079621; doi:10.1186/1479-5876-12-151)
Supplement: Additional file 3: Figure S3 — Growth inhibition of cells treated with IL-4 and the combination of IL-4+TNFα for 3 different RCC cell lines is shown. Same numbers of cells were seeded into wells and cells were equally detached and counted using trypan blue exclusion at the end of culture time (72hrs). Combined data of three different experiments are shown. [file 1479-5876-12-151-S3.pptx]

## Slide 1
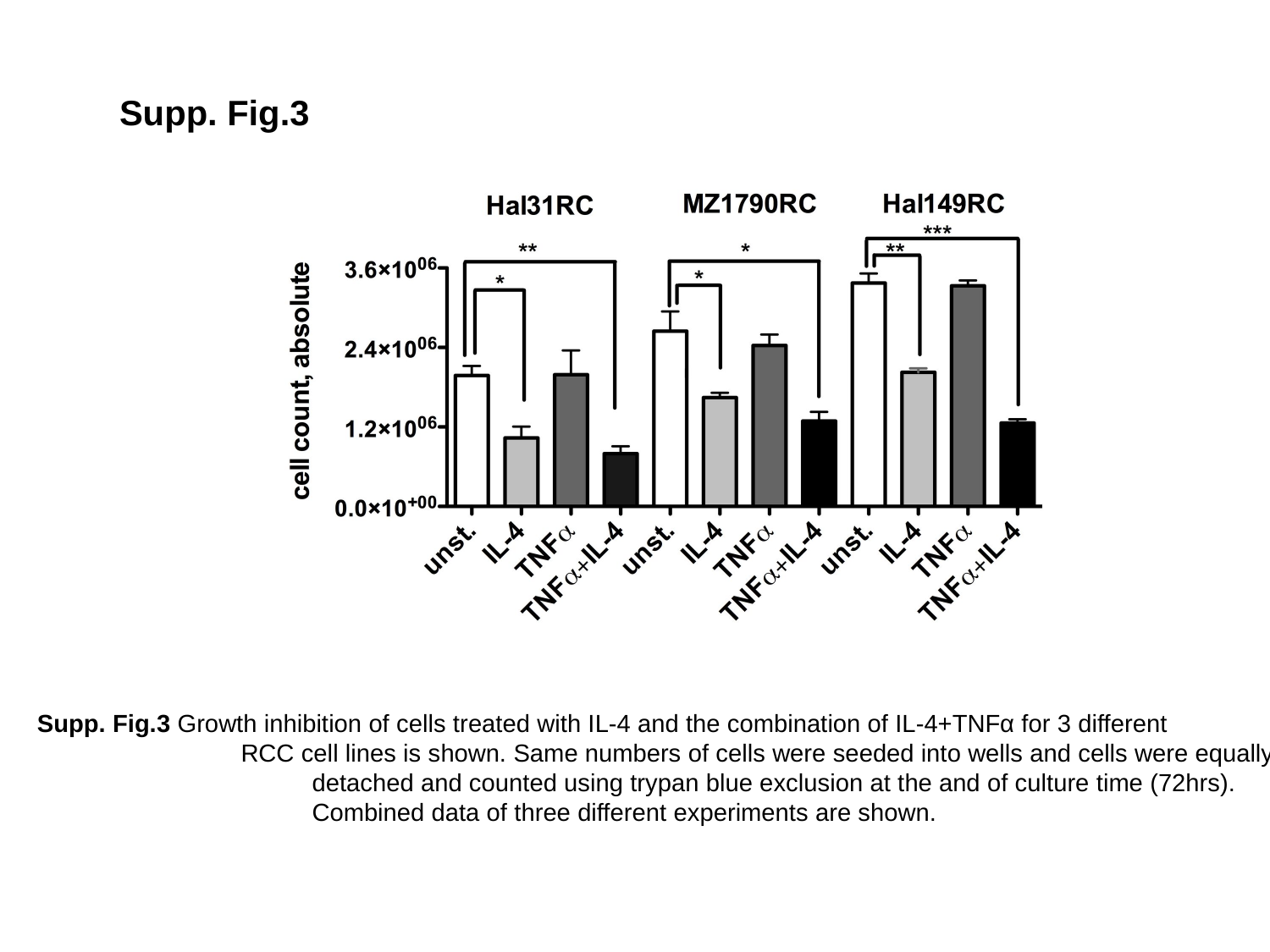

Supp. Fig.3
Supp. Fig.3 Growth inhibition of cells treated with IL-4 and the combination of IL-4+TNFα for 3 different
	 RCC cell lines is shown. Same numbers of cells were seeded into wells and cells were equally
		 detached and counted using trypan blue exclusion at the and of culture time (72hrs).
		 Combined data of three different experiments are shown.
